# Iron Dyshomeostasis in Neurodegeneration with Brain Iron Accumulation (NBIA): Is It the Cause or the Effect?

**DOI:** 10.3390/cells13161376

**Published:** 2024-08-19

**Authors:** Francesco Agostini, Bibiana Sgalletta, Marco Bisaglia

**Affiliations:** 1 Department of Biology, University of Padova, Via Ugo Bassi 58/B, 35131 Padova, Italy; bibiana.sgalletta@phd.unipd.it; 2 Centro Studi per la Neurodegenerazione (CESNE), University of Padova, 35121 Padova, Italy

**Keywords:** autophagy, iron, mitochondria, NBIA, neurodegeneration

## Abstract

Iron is an essential metal ion implicated in several cellular processes. However, the reactive nature of iron renders this metal ion potentially dangerous for cells, and its levels need to be tightly controlled. Alterations in the intracellular concentration of iron are associated with different neuropathological conditions, including neurodegeneration with brain iron accumulation (NBIA). As the name suggests, NBIA encompasses a class of rare and still poorly investigated neurodegenerative disorders characterized by an abnormal accumulation of iron in the brain. NBIA is mostly a genetic pathology, and to date, 10 genes have been linked to familial forms of NBIA. In the present review, after the description of the principal mechanisms implicated in iron homeostasis, we summarize the research data concerning the pathological mechanisms underlying the genetic forms of NBIA and discuss the potential involvement of iron in such processes. The picture that emerges is that, while iron overload can contribute to the pathogenesis of NBIA, it does not seem to be the causal factor in most forms of the pathology. The onset of these pathologies is rather caused by a combination of processes involving the interplay between lipid metabolism, mitochondrial functions, and autophagic activity, eventually leading to iron dyshomeostasis.

## 1. Introduction

Iron is an indispensable nutrient for every cell, and it is involved in essential biological processes such as DNA synthesis and repair, cell proliferation, mitochondrial respiration, and energy production. The adult body typically contains 2–4 g of iron, of which more than 80% is stored in the hemoglobin inside red blood cells [1]. In the brain, iron participates in numerous neuronal processes, including the production and maintenance of myelin and the synthesis of neurotransmitters such as dopamine, norepinephrine, and serotonin [2].

These cellular processes are mediated by several iron-containing enzymes and depend on iron’s ability to cycle between two redox states, ferrous (Fe^2+^) and ferric (Fe^3+^), through the exchange of electrons [2]. However, the reactive nature of Fe^2+^, which makes iron crucial for life, renders this metal ion potentially dangerous for cells. Alterations in iron homeostasis can lead to an overload of free Fe^2+^, which can react with hydrogen peroxide (H_2_O_2_) in the Fenton and Haber–Weiss reactions. The products of these chemical processes are the highly reactive hydroxyl radicals (●HO) that may damage proteins, nucleic acids, and membrane lipids, ultimately leading to cell death [3,4]. In humans, age-related iron buildup is observed in multiple organs, including the liver, kidney, and brain, and this accumulation has been linked to various pathologies such as liver disorders, renal diseases, and neurodegenerative diseases, including Parkinson’s disease [5].

In this review, we will first describe the processes involved in iron metabolism by focusing mainly on the role of mitochondria and lysosomes in its homeostasis and highlighting how alterations in these organelles can be associated with ferroptosis, a cell-death pathway related to iron dyshomeostasis. We will then analyze the involvement of iron in a group of rare and heterogeneous neurodegenerative disorders collectively called Neurodegeneration with Brain Iron Accumulation (NBIA).

## 2. Iron Homeostasis

**Figure 1:**
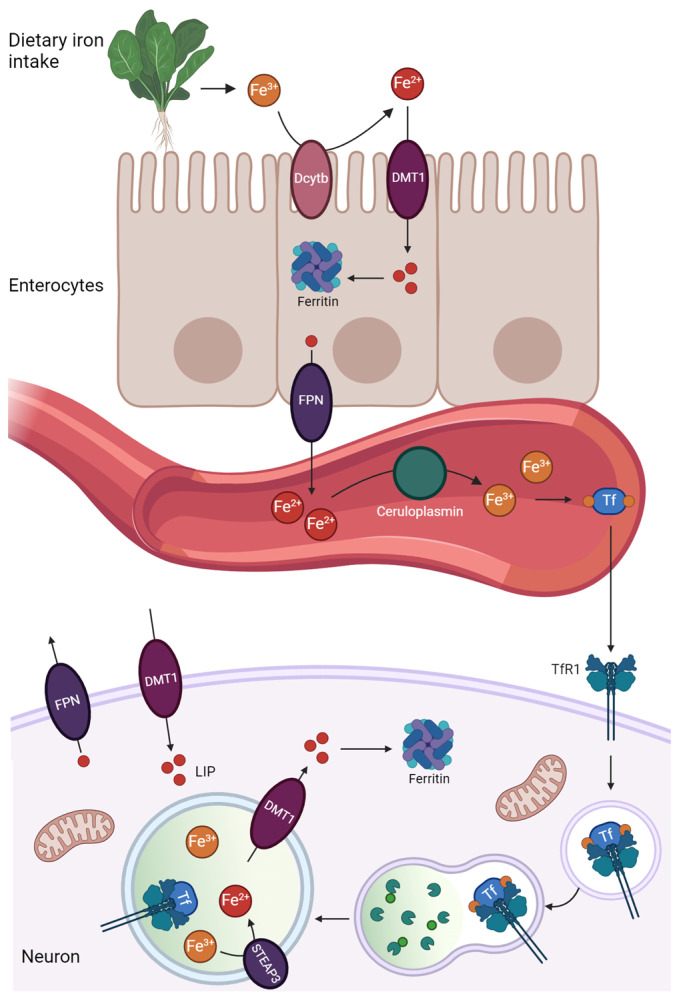
Schematic representation of iron metabolism. Dietary ferric iron is converted to ferrous iron (Fe^2+^) by the duodenal cytochrome B (Dcytb) and absorbed through the divalent metal transporter 1 (DMT1). Here, it can be stored through ferritin or enter the bloodstream by ferroportin (FPN). In the blood, ferrous iron is converted to ferric iron by ceruloplasmin, and in this state, it binds transferrin (Tf). In the brain, the binding of iron-loaded Tf to transferrin receptor 1 (TfR1) allows iron intake through endocytosis within neurons. The acidic lumen of late endosomes and lysosomes allows the release of iron from transferrin. Ferric iron is reduced to ferrous iron by the ferric reductase six-transmembrane epithelial antigen of prostate 3 (STEAP3) and excreted by DMT1 in the cytosol, where it forms the labile iron pool (LIP). Alternatively, DMT1 on the plasma membrane allows free iron to enter neurons. Exceeding intracellular iron is released out of neuronal cells by FPN.

Iron homeostasis is maintained at both systemic and cellular levels, requiring a strict regulation of its metabolism to detect iron levels and fine-tune its absorption and recycling [5]. The principal pathways involved in iron metabolism are summarized in Figure 1.

Dietary iron is absorbed in the small intestine and primarily used for erythrocyte production. Under normal conditions, only 1–2 mg per day of dietary iron is absorbed through the intestine, as this metal ion is typically recycled from senescent red blood cells and then released back into the plasma to produce new erythrocytes [1]. The liver plays a key role in systemic iron homeostasis, acting as a storage site when iron levels in the plasma are excessive and mobilizing iron from hepatocytes into circulation to meet metabolic needs [1]. Unlike iron absorption, controlled mechanisms mediating iron excretion in the human body are not known, and systemic iron excretion occurs at an almost steady basal rate regardless of its concentration in the body [5]. Thus, efficient control of iron uptake is critical for maintaining iron homeostasis.

Dietary ferric iron is reduced in the small intestine by the duodenal cytochrome B to Fe^2+^ and absorbed by the divalent metal transporter 1 (DMT1), localized in the duodenal epithelium. Iron is then transferred into the blood by ferroportin (FPN), a basolateral membrane transporter. After intestinal absorption or export from storage organs, Fe^2+^ in the blood is oxidized back to Fe^3+^ by ceruloplasmin, and in the ferric form, it binds transferrin (Tf). At the microvasculature level of the blood–brain barrier, the interaction between iron-bound Tf and transferrin receptor 1 (TfR1) allows iron to enter the brain by endocytosis and transcytosis within various cell types [6].

The interaction between iron-loaded Tf and TfR1 also mediates iron internalization into neurons via endocytosis. Alternatively, DMT1 on the plasma membrane allows free iron to enter neurons. Other cells, including astrocytes and oligodendrocytes, can internalize iron as low molecular weight complexes, such as iron-citrate or ascorbate, in a way that is independent of the presence of Tf [6]. Intracellularly, iron imported through endocytosis reaches late endosomes and lysosomes, where the low pH (pH~4.5–5.0) reduces Tf affinity for Fe^3+^, causing its release and reduction to Fe^2+^ by the ferric reductase six-transmembrane epithelial antigen of prostate 3 (STEAP3), whose activity depends on the acidic intraluminal environment. Ferrous iron is transported into the cytosol by DMT1, forming the labile iron pool (LIP). Cytoplasmic iron can be used in various cellular processes, delivered to mitochondria, or accumulated in ferritin (Ft), a hollow protein complex composed of 24 subunits with a spherical cage-like structure with an outer diameter of 12~13 nm and an interior cavity diameter of 7~8 nm, which allows the oxidation of Fe^2+^ and its storage as Fe^3+^ [7]. Excess intracellular iron can be released from neuronal cells by ferroportin (FPN), the only known iron exporter (Figure 1) [5,6,8].

### Mitochondria and Lysosomes Participation in Iron Homeostasis

**Figure 2:**
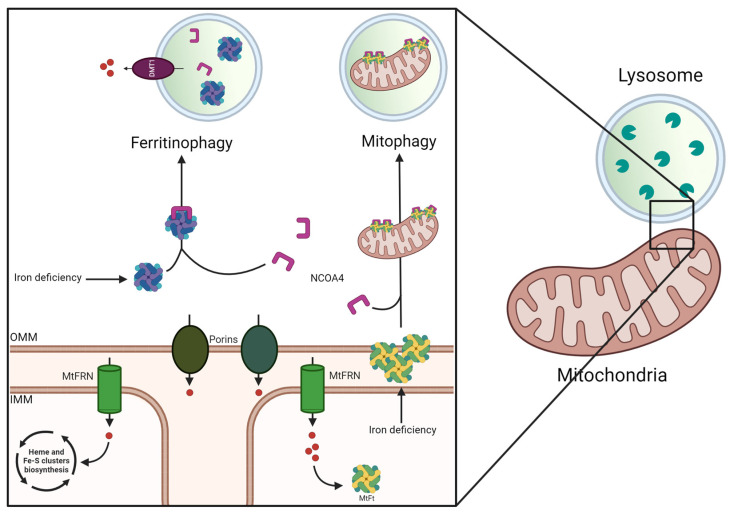
Lysosomal and mitochondrial participation in iron metabolism. Iron uptake in mitochondria is mediated by pore-forming membrane proteins (porins) at the level of the outer mitochondrial membrane (OMM) and mitoferrin (MtFRN) located in the inner mitochondrial membrane (IMM). Within mitochondria, iron is used for the biosynthesis of heme and Fe-S clusters. A portion of mitochondrial iron is stored in the mitochondrial-specific ferritin (MtFt). Upon iron starvation, MtFt accumulates at the level of the OMM, where it binds the nuclear receptor coactivator 4 (NCOA4), thus inducing mitophagy, the process by which entire mitochondria are degraded. Alternatively, when NCOA4 senses low levels of cytosolic iron, it interacts with ferritin (Ft) stimulating its degradation, a process known as ferritinophagy.

Mitochondria are the primary intracellular sites of iron utilization, as represented in Figure 2. In the mitochondrial matrix, iron participates in the biosynthesis of heme and Fe-S clusters, which are cofactors of several proteins involved in fundamental cellular processes such as electron transfer, energy production, and gene expression. These clusters consist of ferrous/ferric iron and inorganic sulfide, enabling electron cloud delocalization over both Fe and S, making Fe-S clusters versatile cofactors [6,9]. Mitochondrial iron uptake is dependent on the presence of mitoferrin (MtFRN). In fact, while pore-forming membrane proteins (porins) allow nonspecific passage of small molecules and ions across the outer mitochondrial membrane (OMM), the inner mitochondrial membrane (IMM) is impermeable to most molecules and ions, which can only reach the mitochondrial matrix through specific membrane-inserted transporters [6]. The mitochondrial matrix contains mitochondrial-specific ferritin (MtFt), which is structurally and functionally similar to its cytosolic counterpart, making mitochondria important sites of iron storage. MtFt acts as a central mediator of low-iron-induced mitophagy, the lysosomal-mediated degradation of mitochondria. Upon iron starvation, mitochondrial damage induces MtFt accumulation on the OMM, where the interaction with the nuclear receptor coactivator 4 (NCOA4) stimulates mitochondria attachment to the growing autophagic phagophore, promoting mitophagy. Thus, under low-iron conditions, mitochondria can become an important intracellular source of iron [9].

Lysosomes also play a crucial role in cellular iron balance (Figure 2). These structurally and spatially dynamic organelles actively perceive both the nutritional and the redox status of cells, generating adaptive responses to maintain cellular homeostasis [10]. Among their numerous cellular functions, lysosomes are also increasingly recognized as master regulators of iron metabolism [9]. Ferritin-associated iron can be released through a specific lysosome-dependent degradation pathway called ferritinophagy. This pathway depends on NCOA4, which senses low cytosolic iron levels. NCOA4 interacts with ferritin and transfers it to the lysosomes for degradation. Thus, the lysosomal breakdown of iron-rich ferritin, along with its mitochondrial counterpart, supplies iron to the cell. Lysosomal ferritin degradation is mediated by various proteases, whose activity depends on the acidic environment of the lysosomal lumen, which is generated and maintained by a transmembrane proton pump. The activity of this pump is crucial for lysosome function, and an impaired lysosomal pH is a hallmark of several disease conditions [11]. For example, loss of lysosomal acidification decreases bioavailable iron levels, promoting mitochondrial dysfunction, oxidative stress, and inflammation. Importantly, dietary iron supplementation can rescue these pathological phenotypes [9]. Since lysosomes mediate the homeostatic degradation of iron-rich ferritin and mitochondria, they can be exposed to elevated iron levels and oxidative stress. To prevent oxidative damage-associated membrane permeabilization, lysosomes need to limit the intraluminal LIP through the formation of complexes with chelating molecules such as cysteine, glutathione, citrate, and polyamines [9].

Overall, the aforementioned considerations emphasize the participation of both mitochondria and lysosomes in maintaining cellular iron homeostasis. In addition, a finely tuned interplay between these organelles plays an essential role in controlling intracellular iron levels. Although cytoplasmic iron import into the mitochondrial matrix is generally mediated by MtFRN on the IMM, mitochondria might also obtain iron from Tf-containing endosomes/lysosomes through transient interactions with these organelles [9]. The formation of membrane contact sites between these organelles has been proposed to be mediated by DMT1 or the voltage-dependent anion-selective channel (VDAC1) localized to both the OMM and endosomal membrane [12]. Similar to recent findings on calcium [13]. These contact sites could mediate direct iron transfer between organelles [14].

## 3. Iron Dyshomeostasis and Ferroptosis

Enhanced iron levels and altered concentrations of iron-related proteins have been detected in different brain regions of elderly people [15]. For instance, higher amounts of ferritin and heme oxygenase-1 (HO-1) were observed in the hippocampus, and cerebral cortex of aged human brains [16], and significant alterations in ferritin heavy and light chains were also detected in brain tissues from Parkinson’s Disease (PD) and Alzheimer’s Disease (AD) patients [17]. Although a direct correlation between brain iron accumulation and age-dependent neurodegenerative processes has not been completely demonstrated, enhanced iron levels are likely linked to increased oxidative conditions and changes in the expression of iron-related genes.

Recently, iron toxicity has been associated with ferroptosis, a regulated cell death mechanism distinct from apoptosis and other types of regulated necrosis [18]. The three main pathological features of ferroptosis are (i) the presence of redox-active iron, (ii) the reduction of the intracellular antioxidant system, particularly glutathione (GSH) and glutathione peroxidase 4 (GPX4), and (iii) the oxidation of polyunsaturated fatty acid (PUFA)-rich membranes. Ferrous iron can promote ferroptosis by mediating the Fenton and Haber–Weiss reactions and/or by stimulating the activity of lipoxygenases, iron-containing enzymes that catalyze the oxygenation of PUFAs, especially arachidonic acid [19,20,21,22]. Besides binding free cytosolic iron in the LIP and preventing its oxidation, GSH acts as a cofactor in the GPX4-mediated reaction, which reduces PUFA-containing hydroperoxides into PUFA-containing alcohols. Since PUFA-rich membrane peroxidation is a key hallmark of ferroptosis, enzymes responsible for PUFA incorporation into membranes have been described to play a crucial role in fueling ferroptosis. Accordingly, Acyl-CoA synthetase long-chain 4 (ACSL4) has been identified as a key component of ferroptosis execution [21]. ACSL4 catalyzes the formation of PUFA-CoA, particularly for arachidonic acid, which is then esterified into glycerol-3-phosphate to generate PUFA-containing phospholipids [21].

The role of mitochondria in ferroptosis is still debated, but evidence emphasizes association with this form of cell death. For instance, cells treated with erastin, which induces ferroptosis through the inhibition of the cellular uptake of the cystine required for GSH synthesis, exhibit increased mitochondrial potential shortly after treatment, followed by mitochondrial depolarization in the next few hours [18,23]. This increased mitochondrial potential is accompanied by ROS production [23], while subsequent mitochondrial depolarization suggests loss of mitochondrial membrane integrity and alterations in mitochondria functionality [24]. In addition to changes in the membrane potential, mitochondrial morphological alterations are observed, comprising an increased membrane density and volume reduction [18,25]. Notably, morphological hallmarks typical of other forms of cell death, such as necrosis (cytoplasmic swelling, plasma membrane rupture), apoptosis (chromatin condensation and margination), or autophagy (formation of double-membrane enclosed vesicles) have not been described in the erastin-promoted ferroptosis [26]. Significant mitochondrial morphological and functional alterations have also been observed in cells treated with RSL3, another molecule that induces ferroptosis by inhibiting GPX4 activity [26]. The role of mitochondria in ferroptosis is further supported by a cellular model based on cysteine deprivation. Since both the tricarboxylic acid cycle (TCA) and the electron transport chain (ETC) activity of mitochondria have been shown to promote ferroptosis, the participation of these organelles in ferroptosis was proposed to depend on their metabolic functions [27]. More recently, in an erastin-based cellular model, loss of mitochondrial membrane potential and depletion of intracellular ATP have been observed alongside ROS accumulation over time [28]. Interestingly, cell treatment with cyclosporine A, which inhibits the activation of the mitochondrial permeability transition pore (mPTP), also prevents cell death. Cyclosporine A prevents ROS accumulation only in later stages (48 h) but is almost ineffective at earlier time points (4 and 24 h), suggesting mitochondrial damage may be the terminal process of ferroptosis [28].

Given their role in iron homeostasis, lysosomes are also closely linked to ferroptosis. Although ferroptosis has been initially described as a non-autophagic form of cell death [18], the participation of autophagy and lysosomal activity in ferritin degradation supports the role of the autophagic-lysosomal pathway in this process. NCOA4-mediated lysosomal degradation of ferritin and/or mitochondria increases cytosolic free iron levels, which ultimately can fuel ferroptosis. Moreover, the lysosomal accumulation of redox-active iron has been suggested to promote ferroptosis through the peroxidation of the lysosomal membrane lipids and the subsequent permeabilization of the lysosomal membrane [29,30,31]. This can enhance the production of lipid radicals, further propagating oxidative damage and cell membrane rupture [29,30,31]. Accordingly, the inhibition of ferritinophagy through NCOA4 knockdown or autophagy blockage has been shown to hamper free iron accumulation [32,33].

Importantly, this subtype of regulated cell death has been linked to the occurrence and development of several human diseases, such as stroke, intracerebral hemorrhage, ischemia-reperfusion injury, acute kidney failure, traumatic brain injury, and neurodegenerative diseases, including PD, AD, and Friedreich’s ataxia [33]. Considering the role of iron in the regulation of ferroptosis, it is not surprising that this process is particularly relevant in the context of a class of neurodegenerative disorders characterized by the accumulation of this metal ion. These pathologies are grouped together under the name of neurodegeneration with brain accumulation of iron (NBIA).

## 4. NBIA, Clinical and Histopathological Aspects

In 1922, the observation of basal ganglia iron deposition in a small group of people with escalating dysarthria and dementia led to the definition of a new disease, originally known as Hallervorden–Spatz syndrome, from the name of the clinicians that first described it [34]. Nowadays, NBIA collectively refers to the spectrum of neurodegenerative disorders characterized by brain iron inclusions, mainly in the *globus pallidus* and *substantia nigra* [35]. NBIA is extremely rare; it targets about two people per million individuals in the general population and affects both children and adults [36]. The onset usually occurs before 6 years of age, and it is followed by a rapid progression. However, atypical forms of NBIA with late onset and/or slower progression have also been observed [37]. NBIA is clinically heterogeneous, and in most cases, the first symptoms are represented by dystonia and dysarthria, followed by spasticity, chorea and parkinsonism that lead to the loss of ambulation capacities within fifteen years after the onset. Oculomotor impairments, pigmentary retinopathy, and cognitive features may also occur [38,39]. In addition, most patients experience gastroesophageal reflux and constipation, and they often require tube feeding in the later stages of the disease due to swallowing difficulties. Malnutrition and aspiration pneumonia are secondary complications that usually lead to death [37].

By definition, NBIA disorders are characterized by high levels of iron in the brain, as confirmed through magnetic resonance imaging (MRI) and post-mortem examination [40]. This iron accumulation is mainly found in microglia, macrophages, and neurons; moreover, it has been observed in the extracellular space, especially around blood vessels [37,41].

In all NBIA subtypes, the highest concentration of iron is found in the globus pallidus [39], one of the subcortical basal ganglia structures in the brain coordinating voluntary and proprioception movements [42]. The substantia nigra pars reticulata is another area of the brain with a significant increase in iron concentration in NBIA cases, even though this brain region often appears to be affected in the later stages of the disease [43]. Neuroferritinopathy and aceruloplasminemia are the only NBIA subtypes in which significant iron accumulation also occurs in the liver and in other areas of the brain, such as the striatum, dentate nuclei, thalamus, and red nuclei [36].

Besides iron accumulation, one of the features of the NBIA spectrum is the presence of spheroid bodies in the central nervous system (CNS), primarily at the level of globus pallidus and the surrounding structures [39]. Spheroid bodies refer to degenerating axons that assume a particular distended and swelling morphology [44]. In addition, α-synuclein positive Lewy bodies and tau-containing neurofibrillary tangles represent additional histological features found in *substantia nigra pars compacta* and cortex of most NBIA cases [45]. These inclusions are observed mostly in subjects with protracted clinical course, suggesting that protein aggregation occurs in the later stages of the disease [37].

## 5. NBIA: Genetic and Physiopathological Aspects

Although approximately 15–20% of NBIA cases are idiopathic, in most of the NBIA-affected patients, the pathology has a clear genetic origin, and NBIA subtypes are classified according to their genetic basis [36,46]. To date, 10 genes have been linked to the familial forms of NBIA [34]. The majority of them are transmitted in an autosomal recessive way, although in some of them, an autosomal dominant or X-linked mechanism of inheritance has been observed [47]. Although NBIA pathogenesis is still poorly understood, and the physiological functions of some of the proteins associated with the genetic forms of the pathology have not been elucidated to date, their study gave us some important hints to better understand the main cellular processes underlying this complex pathology [37].

As aforementioned, NBIA encompasses a family of neurodegenerative disorders, sharing the accumulation of brain iron as a characteristic hallmark. At the cellular level, they show heterogeneous phenotypes, reflecting the genetic differences at the basis of these disorders [36]. Indeed, the genes associated with the genetic forms of NBIA can be classified into four subtypes according to the main intracellular processes primarily impacted by the disease. They comprise NBIA affecting (i) iron homeostasis, (ii) lipid metabolism, (iii) mitochondrial function and (iv) autophagic activity. These classes of NBIA are summarized in Table 1 and will be described in the following paragraphs.

### 5.1. NBIA Associated with Iron Homeostasis

The correlation between iron overload and NBIA confirms that the maintenance of the correct iron level is key for cell viability, specifically in the brain [48]. Surprisingly, just two of the NBIA-associated genes contribute to the maintaining of iron homeostasis, and they are associated with the two NBIA forms characterized by the most widespread iron deposition. Thus, even though iron accumulation is a gold standard for NBIA diagnosis, it is still not clear whether it is the main cause or a consequence of other cellular impairments leading to neurodegeneration.

Among the proteins linked to the genetic forms of NBIA and involved in iron homeostasis, ceruloplasmin is a ferroxidase that catalyzes the oxidation of Fe^2+^ to Fe^3+^, participating in the trafficking of iron and facilitating its translocation from and to the cell [49]. Importantly, mutations of the *Ceruloplasmin* (*CP*) gene cause a form of NBIA called Aceruloplasminemia, a very rare disorder with an estimated prevalence of one in two million [50]. More than 60 mutations in the gene have been described to date; all of them cause the complete loss or a strong reduction of ceruloplasmin activity. The vast majority of patients have homozygous mutations that are inherited in an autosomal recessive fashion, but compound heterozygous mutations have also been reported [50]. The downregulation or absence of the protein function leads to iron accumulation, as demonstrated in the post-mortem brains of patients carrying these mutations [51,52]. Importantly, the iron overload distinctive of this form of pathology leads to a series of cascading effects resulting in oxidative stress and lipid peroxidation in brain tissues [53,54]. Coherently, in vitro analysis confirmed that ceruloplasmin loss-of-function causes the increase of ROS as well as cell death [54]. These phenotypes are primarily restricted to the basal ganglia, thalamus and cerebellum both in human patients and in *CP* KO mouse models [53], highlighting the fact that some areas of the brain are particularly susceptible to impairments in iron metabolism and likely less efficient in restoring the homeostatic conditions. This might explain why the basal ganglia represent the primary site for neurodegeneration associated with iron overload.

The *ferritin light chain* (*FTL*) is the other NBIA-associated gene with a role in iron homeostasis, and mutations of this gene are causative of a form of NBIA called neuroferritinopathy, an autosomal dominant disease that has been diagnosed in less than 50 people worldwide to date [47,55]. The gene encodes for one of the ferritin subunits that improves the stability and, therefore, the function of the protein [55,56]. Nine mutations have been described so far; all of them affect the c-terminal region of the protein, resulting in alterations of ferritin structure, preventing its binding with iron and its iron storage properties [57,58]. The activity of ferritin is fundamental to avoiding iron accumulation and the consequent increase of ROS production and lipid peroxidation. In vitro analysis performed in patient-derived fibroblasts demonstrated that neuroferritinopathy causes an increase in ROS and the reduction of the transferrin receptor levels. Similarly, murine models showed that ferritin function impairments lead not only to increased iron levels, overproduction of ROS, and lipid peroxidation but also ferritin aggregation, which may indicate defects in its degradation [59].

In both these NBIA subtypes, iron accumulation appears to be the triggering event for the disease, demonstrating once again the importance of iron regulation for the preservation of cell homeostasis. The two forms of NBIA reported in this paragraph also give us some hints about one of the hallmarks of NBIA, which is represented by the increase in lipid peroxidation. As it will be stated throughout the following sections, lipid peroxidation is observed in most of the forms of NBIA, suggesting the crucial participation of lipids in these pathologies.

### 5.2. NBIA Associated with Lipid Metabolism

Lipids, in particular phospholipids, sphingolipids and cholesterol, are the main constituent of biological membranes, participating in the regulation of their structure, rigidity and permeability [60]. Even though lipids are crucial for cell functions, to date, their participation in processes leading to neurodegeneration is much less understood. Nevertheless, several indications support the association between the impairment in lipid homeostasis and neurodegenerative diseases, such as the presence of alteration in lipid metabolism and concentrations observed in PD patients [61] or the association between *GBA1*, which is crucially involved in the metabolism of glycolipids and PD [62]. NBIA constitutes another clear example of the involvement of lipids in neurodegeneration since lipid dyshomeostasis can be included among the shared features of these disorders. Moreover, some of the NBIA-associated genes appear to be directly involved in lipid metabolism.

The *phospholipase A2 group* VI (*PLA2G6*) is one of the NBIA-associated genes linked to lipid homeostasis. About 200 autosomal recessive mutations of this gene have been reported to be the cause of PLA2G6-Associated Neurodegeneration (PLAN), a rare syndrome affecting less than one subject per million people [47,63,64]. The pathology is caused by the loss of function of the protein encoded by *PLA2G6*, the calcium-independent phospholipase A2 beta (iPLA2β) [63]. This enzyme catalyzes the hydrolysis of membrane phospholipids with the generation of free fatty acids and lysophospholipids, being essential for both lipid metabolism and the regulation of biological membrane integrity [65,66]. Besides participating in the definition of membrane phospholipid composition, iPLA2β is also involved in sphingolipid metabolism [66]. Sphingolipids are abundant in the nervous system and have been correlated to neurodegenerative disorders, including PD and AD [66]. 

Moreover, alterations in sphingolipid metabolism have been suggested to promote Tau and Aβ accumulation [66]. Accordingly, *PLA2G6* KO mice models showed that the absence of the protein not only alters the metabolism of phospholipids but also causes the abnormal accumulation of ubiquitinated proteins in the brain [67]. Similarly, the analysis of several animal models and human patients demonstrated the accumulation of cytosolic aggregates containing ubiquitinated proteins, such as α-synuclein and phosphorylated tau [63]. These data suggest that defective degradation activity and autophagic dysfunctions may contribute to determining the pathological features of NBIA [67]. In addition, mitochondrial impairments have been observed in *Drosophila* iPLA2β loss of function models as well as in fibroblasts obtained from PLAN patients [68]. These defects comprise an increased lipid peroxidation within mitochondria that alters membrane composition and properties. Moreover, these models display modifications of mitochondrial morphology and dynamics, with respiratory chain dysfunctions and reduced ATP production [68].

Another NBIA-gene with a role in lipid metabolism is *FA2H* (*fatty acid 2-hydroxylase*), whose mutations lay at the basis of Fatty Acid Hydroxylase-Associated Neurodegeneration (FAHN) [47], a rare autosomal recessive disease with an estimated prevalence lower than one in one million [69]. About 65 loss of function mutations have been found in the *FA2H* gene, which encodes an NADPH-dependent mono-oxygenase. This protein is crucial in the generation of 2-hydroxy sphingolipids, a subset of sphingolipids that contain 2-hydroxy fatty acids, whose synthesis is essential for lipid-lipid interactions within cellular membranes [70]. Besides ensuring the physiological shaping of biological membranes, FA2H also participates in myelin formation and is crucial during brain development [71].

Accordingly, demyelination and degeneration of neuronal membranes represent the main features of FAHN, as exemplified by *FA2H* KO mice models [72]. Furthermore, a *Drosophila* model for FAHN demonstrated that at the cellular level, the loss of the FA2H function alters the autophagic machinery, as suggested by the accumulation of the autophagosomal marker LC3, and affects mitochondrial morphology, reducing the connectivity of the mitochondrial network [73]. Notably, these results were confirmed also in human patient fibroblasts [73]. These data demonstrate that lipid metabolism and mitochondrial and autophagic activities are highly interconnected in NBIA, suggesting that these three factors, when altered, may contribute to the onset and progression of the disease.

#### Lipid and Iron Crosstalk in NBIA

Lipids and iron are tightly interconnected, supporting the notion that they can both concur to exacerbate the NBIA pathological hallmarks and lead to neurodegeneration. The effects of iron on lipid homeostasis have been characterized from both a physiological and pathological viewpoint [74]. For instance, iron plays an essential role in myelin synthesis and maintenance [75]. At the same time, lipid peroxidation is one of the main cellular mechanisms underlying iron toxicity [76,77]. Importantly, as already stressed, this process is one of the main features of NBIA, observed in every subtype of these disorders. As previously mentioned, lipid peroxidation involves the action of a free radical on lipids, determining the final addition of oxygen in lipids containing carbon-carbon double bonds. This leads to the cyclic formation of lipid peroxyl radicals and lipid radicals. Iron, in its redox-active state, can directly promote lipid peroxidation [20,76]. Moreover, lipoxygenases, which are involved in the catalytic oxygenation of PUFAs, have been demonstrated to depend on iron for their activity [19,21,22]. Lipid peroxidation has been associated not only with the increase of membrane rigidity and the reduction of membrane-bound protein activity but also with alterations of important biological processes, such as signal transduction, gene expression, substrate–receptor interaction and ferroptosis induction [20,78]. While iron accumulation and its effects on lipid peroxidation can explain the negative consequences at the intracellular level, eventually leading to cell death and neurodegeneration, a direct effect of lipid dyshomeostasis on iron level regulation is less obvious. Nevertheless, considering the key action of phospholipids in the modulation of biological membrane dynamics, it is easy to hypothesize that alterations of lipid homeostasis can promote a cascade of effects affecting iron metabolism and levels. For instance, alterations in lysosomal and/or mitochondrial membrane permeability may impact their iron storage properties, leading to the accumulation of redox-active iron in the cytosol. In this frame, the aforementioned physiological functions of PLA2G6 and FA2H suggest that the iron accumulation detected in these forms of NBIA may represent a secondary effect indirectly derived from lipid dyshomestasis.

### 5.3. NBIA Associated with Mitochondrial Function

Mitochondria are frequently regarded as the powerhouses of the cell due to their function as energy generators in the form of ATP [79]. However, their role goes far beyond that since they are cellular hubs that can influence different pathways and processes, such as autophagy and apoptosis [79]. For this reason, the conservation of mitochondrial quality control is essential to guarantee a balanced cellular homeostasis. In this scenario, it is not surprising that mitochondrial impairments are frequently associated with human diseases and are linked to neurodegeneration [80,81].

Among the NBIA subtypes, the most common form of the pathology is caused by mutations in the *pantothenate kinase 2* (*PANK2*) gene, which is responsible for the so-called Pantothenate Kinase-Associated Neurodegeneration (PKAN), a subtype of the disease with an estimated prevalence of three in one million [36,82]. More than 120 autosomal recessive mutations in this gene have been described as causing the reduction or complete loss of function of the PANK2 protein. Nevertheless, some mutations are associated with defects in PANK2 dimerization or loss of the mitochondrial targeting sequence, resulting in the cytosolic localization of the protein, suggesting that any type of disruption of this kinase function can lead to pathologic phenotypes [83,84]. This enzyme catalyzes the ATP-dependent phosphorylation of pantothenate, an essential regulatory step in Coenzyme A (CoA) biosynthesis. PANK2 also acts as a sensor of mitochondrial CoA and is tightly regulated by the level of CoA and its thioesters through a negative feedback mechanism [85]. In the pathological context, this sensing function is not controlled and leads to energy impairments, ROS production and apoptosis [85]. Moreover, mice models for PKAN display mitochondrial membrane potential defects, increased mitochondrial ROS and enhanced iron levels [86]. In addition, lipid peroxidation, mitochondrial respiration deficits and premature cell death have been detected in neuronal cells generated from induced pluripotent stem cells (iPSCs) from PKAN patients [87,88].

Importantly, CoA is strongly related to lipid homeostasis since it is key in the metabolism of fatty acids, and its synthesis depends on the β-oxidation of triglycerides. Accordingly, the deregulation of this molecule results in lipid dyshomeostasis, including reduced levels of different lipids such as triglycerides, cholesterol metabolites and sphingomyelins, and impaired biosynthesis and replacement of phospholipids in cell membranes [89]. These data emphasize once again how alterations of processes involved in different subtypes of NBIA, such as mitochondrial activity and lipid metabolism, are not independent but rather highly interconnected, and their impairments can contribute to the development of these diseases.

Similar to the pantothenate kinase, CoA synthase plays a role in the CoA generation, catalyzing the last two steps of the biosynthesis of this molecule, and is predominantly located in the mitochondrial matrix [90]. This protein is encoded by the CoA synthase (*COASY*) gene, whose mutations result in the NBIA subtype called COASY Protein-Associated Neurodegeneration (CoPAN), a rare autosomal recessive disease occurring in less than one in every million people [36]. Importantly, the complete loss of function of the protein results in prenatal death; accordingly, the pathological variants cause the instability of the protein and the reduction of its level [91]. Mutant yeast lines of CoPAN models show increased sensitivity to H_2_O_2_, confirming that oxidative stress is one of the main features observed in several of the NBIA forms [92]. Moreover, the absence of CoA synthase also causes decreased oxygen consumption, mitochondrial respiratory deficiency, and increased levels of iron and lipid metabolism abnormalities [92]. Once again, these data underline the strict correlation between mitochondrial defects and lipid dyshomeostasis.

Another gene linked to NBIA that has a mitochondrial function is *C19orf12*, whose mutations cause Mitochondrial Membrane Protein-Associated Neurodegeneration (MPAN), a rare form of the disease with an estimated prevalence of less than one in one million [93,94]. Interestingly, MPAN is considered an autosomal recessive disorder, and around 50 mutations have been reported; both homozygous and compound heterozygous mutations have been found. Nevertheless, monoallelic variants that have a dominant negative effect on the wild-type allele have been observed [95]. *C19orf12* encodes a small transmembrane protein of 17 kDa, which is widely expressed in the brain and localizes in the mitochondria membranes and in the ER [96]. 

On the contrary, pathogenic mutant proteins have been observed to lose their membrane localization, likely preventing the protein from exerting its physiological function [97]. Although the function of C19orf12 is still elusive, in Hela cells, the protein has been demonstrated to respond to oxidative stress insults by changing its intracellular localization and forming aggregates at the mitochondrial level, suggesting its putative role in ROS sensing [97]. Fibroblasts from MPAN patients display mitochondrial fragmentation, reduced oxygen consumption rate and increased ferroptosis [96]. Autophagy alterations were also observed, with decreased levels of the LC3 lipidated form [98]. Accordingly, postmortem brain examination of patients with MPAN revealed not only iron accumulation but also the presence of α-synuclein-positive Lewy bodies and hyperphosphorylated tau-containing inclusions, suggesting defects in the degradative pathways [98]. In addition, a recent *Drosophila* MPAN model supported the association of the pathology with lipid metabolism, including a reduction of triglyceride stores and increased lipolysis [99]. Overall, the studies of C19orf12 further support the association between mitochondria, autophagic function and lipid homeostasis in NBIA onset and progression.

#### Mitochondria and Iron Homeostasis in NBIA

As previously described, mitochondria represent one of the principal intracellular sites of iron utilization. This suggests that mitochondrial function is highly dependent upon a strict regulation of iron levels. Therefore, it is not surprising that alterations in intracellular iron concentrations may have a strong impact on mitochondrial dynamics.

In the same way, the relatively high concentration of iron within mitochondria and their role as iron-storage sites makes these organelles key elements in the control of the amount of intracellular iron. Similarly, mitophagy perturbations may have a strong impact on the iron recycling processes, affecting the homeostasis of this metal ion. This further confirms the important role of the autophagic-lysosomal pathway in iron homeostasis, which is also supported by the presence of NBIA-associated genes linked to the autophagic function.

### 5.4. NBIA Associated with Autophagy

Autophagy is the major catabolic process that allows the degradation of cell material, such as misfolded proteins or damaged, dysfunctional organelles [100]. Autophagic activity is particularly crucial in neuronal cells, whose survival depends upon a strict protein and organelle quality control as they cannot rely on cell division to dilute the toxic effects of an abnormal accumulation of cell debris [101]. Coherently, the link between autophagic dysfunction and neurodegeneration has been demonstrated and widely investigated [102,103]. As already mentioned, the key role of autophagy in NBIA is confirmed by the fact that two of the genes associated with the genetic forms of this pathology have a function in the autophagic pathway.

The first of these genes is *WD repeat domain 45* (*WDR45*), which is localized in the X chromosome and encodes the WDR45 protein. WDR45 belongs to the WD40 repeat protein family with a β-propeller platform structure, and the NBIA form associated with mutations of this gene is called β-propeller protein-associated neurodegeneration (BPAN). More than 100 mutations in *WDR45* have been reported; they are transmitted in an X-linked dominant manner and cause the loss of protein function [104]. The β-propellers are structural motifs that are mainly involved in protein–protein interactions [105]. Coherently, WDR45 participation in the autophagic machinery depends on its binding with phosphatidylinositol-3-phosphate to regulate autophagosome formation, expansion and closure [106]. Accordingly, mutations leading to the loss of function of this protein in human fibroblasts result in autophagic flux dysfunction as well as accumulation of autophagic substrates, like p62, ubiquitinated proteins and protein aggregates [107]. In the same model, mitochondrial alterations were also observed, including decreased mitochondrial branching and network connectivity [97]. In addition, *WDR45* knock-out mice display accumulation of iron in the basal ganglia, together with increased apoptosis and neuronal degeneration [108].

The second NBIA gene associated with the autophagic function is *ATP13A2*. About 50 mutations in this gene have been described to cause an autosomal recessive rare form of NBIA called Kufor–Rakeb disease (KRD) [47,109]. *ATP13A2* synthetizes a 5P-type ATPase, which is mainly localized at the lysosomal level but is also present in endosomes and autophagosomes [110]. The protein seems to participate in the lysosomal homeostasis of ions, such as zinc, manganese and iron, allowing the active transportation of these elements across the lysosomal membrane [111]. In addition, the ATP13A2 protein has been recently implicated in the active transport of polyamines from the endo-lysosomal compartments to the cytosol [109]. The decrease in protein activity results in the impairment of autophagic flux, which causes the accumulation of insoluble proteins and dysfunctional organelles [112]. Moreover, both human fibroblasts and *C. elegans* models for KRD demonstrated that ATP13A2 function participates in mitochondrial homeostasis and that its absence results in mitochondrial fragmentations and an increase of mitochondrial ROS [113]. These phenotypes can ultimately lead to neuronal apoptosis and neuroinflammation [113].

#### 5.4.1. Autophagy and Iron Homeostasis in NBIA

As previously emphasized, the correlation between autophagic defects and neurodegeneration is well established, and mutations in genes involved in autophagy can be linked to iron accumulation in NBIA. In fact, as explained in the previous sections, lysosomes play a key role in the storage and recycling of iron. This function makes these organelles highly relevant in the intracellular regulation of iron metabolism.

The autophagic-mediated degradation of ferritin that may be intensified by iron overload has also been associated with ferroptosis [32,114] and could be directly related to NBIA pathogenesis. Importantly, iron contained in the lysosomal lumen is mainly redox-active. Indeed, the acidic pH of lysosomes promotes the reduction of ferric to ferrous iron [115]. In this redox state, in normal physiological conditions, iron is rapidly released into the cytoplasm and recycled to sustain cell homeostasis [116]. In this scenario, the unregulated increase of iron levels within lysosomes can be highly detrimental, as these organelles are very susceptible to oxidant-mediated destabilization. For example, the Fenton and Haber–Weiss reactions leading to lipid peroxidation of the lysosomal membrane can cause the membrane permeabilization of the organelle. Lysosomal membrane permeabilization may, in turn, result in the leakage of the lysosomal content, including lytic enzymes, eventually leading to apoptosis or necrosis [29,31,32]. Overall, the function of lysosomes as iron storage and their intrinsic susceptibility to iron-mediated oxidative reactions may explain how iron homeostasis and lysosomal function can influence each other and, in NBIA-related pathological conditions, be mutually detrimental.

#### 5.4.2. Mitochondria–Lysosomes Interplay in Iron Metabolism

As highlighted throughout the previous paragraphs, lipids, mitochondria and lysosomes seem to play a central role in all the reported NBIA forms. This clearly suggests that these three factors are mutually susceptible to iron intracellular dyshomeostasis and, at the same time, highly interconnected in promoting alterations in iron levels. While the interplay between lipid metabolism and mitochondrial or lysosomal function in iron regulation has been described in the previous sections, the effects of the mitochondria–lysosome crosstalk in iron homeostasis have been poorly investigated and characterized. It is well-known that mitochondria and lysosomes can employ a variety of communication mechanisms to finely tune each other functions, and these mechanisms could also be relevant in regulating iron metabolism. The most characterized is the autophagic-dependent mitochondrial degradation through mitophagy [117]. The rate of mitochondrial degradation can be affected by the levels of iron, as iron loss due to iron chelation has been observed to trigger mitophagy [118]; in turn, mitochondrial autophagy can influence the lysosomal storage and the recycling of this metal ion. Another mechanism of mitochondria–lysosome crosstalk is represented by physical contact. As already mentioned, it has been shown that mitochondria–lysosome contact sites can promote the transfer of iron from one organelle to the other. This may be a way to easily and rapidly regulate the iron concentration at the level of both organelles. Nevertheless, this process is still largely unknown, and a fine characterization of the physiological role of this direct transport of iron, as well as the molecular mechanisms behind this process, would be extremely relevant for a better comprehension of the physiopathological mechanisms underlying NBIA.

A third mechanism that allows the interplay between mitochondria and lysosomes is through long-distance communication, ensured by the regulation of intracellular signaling pathways that can transduce the signals coming from one organelle and send them to the other [119]. In this framework, it would be very informative to investigate whether the organelle(s) concentration of iron can be sensed by specific proteins that can integrate this information into other sites of the cell. For instance, the AMP-activated protein kinase (AMPK) has already been demonstrated to participate in the communication between mitochondria and lysosomes [120]. Even though AMPK function has not been directly associated with iron, the activation of the protein, which largely depends on mitochondrial activity and is known to promote autophagy, has been recently linked to the inhibition of ferroptosis [121]. The molecular mechanisms behind this AMPK function are still poorly understood, but they seem to rely on the AMPK participation in the acetyl-CoA pathway by inhibiting fatty acid synthesis, which prevents lipid peroxidation and, therefore, ferroptosis [121]. This role of AMPK suggests that there may be some sort of long-distance communication between mitochondria and lysosomes that can modulate the metabolism of iron or its effect on cell homeostasis.

Overall, the investigation of the effect of iron metabolism in the interplay between mitochondria and lysosomes would greatly increase our knowledge of the mechanisms behind iron dyshomeostasis in general and, more specifically, in NBIA.

### 5.5. NBIA Associated with DCAF17

One of the rarest forms of NBIA, referred to as Woodhouse–Sakati syndrome, is an autosomal recessive disease linked to mutations in the *DCAF17* gene [122]. Thirteen variants have been described to date, and the prevalence of the disorder has been estimated to be less than one in one million [123]. *DCAF17* gene encodes a nucleolar protein of unknown function, which seems to affect apoptosis, DNA methylation and cell cycle progression [122]. Even though the function of this protein does not appear to be related to the molecular pathways analyzed in this review, further investigations are required for a better definition of its physiological role and its involvement in NBIA pathology.

## 6. Discussion and Conclusions

**Figure 3:**
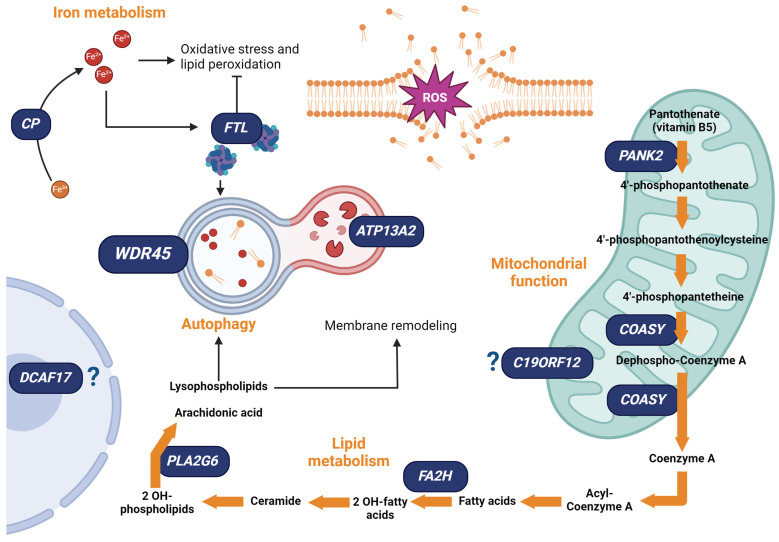
Schematic representation of the cellular pathways involving the NBIA-associated proteins. Ceruloplasmin (CP) and ferritin light chain (FTL) directly participate in iron metabolism. CP catalyzes the oxidation of ferric iron (Fe^2+^) to ferrous iron (Fe^3+^) and is responsible for iron transfer through the plasma membrane. The *FTL* gene encodes a subunit of ferritin, which acts as an iron storage protein, avoiding its accumulation in the cytosol. At the mitochondrial level, pantothenate kinase (PANK2) catalyzes the phosphorylation of pantothenate, the first step of Coenzyme A biosynthesis. Coenzyme A synthase (COASY) catalyzes the last two steps of Coenzyme A synthesis, while the function of C19orf12 is still largely unknown. The proteins fatty acid 2-hydroxylase (FA2H) and Phospholipase A2 group 6 (PLA2G6) participate in lipid metabolism. FA2H is responsible for the hydroxylation of fatty acids, which are essential for the synthesis of ceramides. Ceramides are the precursors of dihydroxy phospholipids, key components of cell membranes. Phospholipase A2 group 6 (PLA2G6) catalyzes the hydrolysis of membrane phospholipids, releasing arachidonic acid and lysophospholipids, which are involved in the regulation of several cellular processes, such as membrane remodeling and autophagy. The proteins WDR45 and ATP13A2 play a role in the autophagic pathway. WDR45 regulates autophagosome formation, and ATP13A2 is a lysosomal ATPase that is involved in cation and polyamine transport through the lysosomal membrane. Finally, DCAF17 is a nucleolar protein with still unknown functions. All the mentioned pathways are somehow associated with iron metabolism, and their dysfunction induces cytosolic iron accumulation with the generation of oxidative stress conditions and lipid peroxidation, which eventually lead to cell damage and cell death.

NBIA encompasses a class of heterogeneous neurodegenerative, still incurable disorders that have as a common denominator the accumulation of iron in the brain. At present, 10 genes have been associated with this pathology (Figure 3). While the analysis of the different genetic forms of NBIA allowed the definition of cellular pathways potentially involved in the pathogenesis, several unanswered questions need to be considered for a better understanding of the disease and the definition of therapeutic strategies.

A crucial aspect that needs to be tackled is the precise role of iron in NBIA pathology. While its involvement in NBIA-related pathological processes is obvious, it has not been established yet whether iron accumulation is the primary cause or a consequence of the cellular processes mainly affected by NBIA. The analysis of the cellular mechanisms associated with the different NBIA subtypes described in this review leads us to suggest that iron deposition represents one of the characteristic features rather than a common cause of the pathological phenotype. In fact, besides neuroferritinopathy and aceruloplasminemia, in which iron dyshomeostasis appears as the triggering factor of the pathology, the other NBIA subtypes are more likely elicited by other factors. Alterations in lipid metabolism and/or mitochondrial and lysosomal defects could represent causal events in the disease onset and progression, leading to iron overload as a consequential effect. However, since all these processes mutually influence each other, further investigation is required to definitely resolve this issue.

Partially related to the previous question, it would be crucial to precisely understand why neurons of the basal ganglia are mostly susceptible to iron overload. Noteworthy, even in physiological conditions, this brain region is characterized by an age-dependent accumulation of iron [124], suggesting that either this metal ion may have a specific role in the functioning of basal ganglia nuclei or specific mechanisms are responsible for iron accumulation in these brain structures.

In conclusion, even though the investigation on NBIA still needs to be expanded, in the last few years, the exploitation of novel models for the genetic forms of the pathologies has been giving us great opportunities to increase our knowledge about this form of neurodegeneration. By following this path, scientific research could lead to the discovery of novel therapeutic approaches for this still incurable class of disorders and give hope to many people suffering from this type of neurodegeneration.

## Figures and Tables

**Table 1:** The familial forms of NBIA. The pathologies are subdivided by colors according to the main cell process they are implicated in. Iron homeostasis (red); lipid metabolism (blue); mitochondrial function (yellow); autophagic activity (green). Woodhouse–Sakati syndrome (grey) does not fit into these classes.

**Gene** **(Protein)**	**NBIA Form**	**Protein Function**	**Cellular Phenotype**
*Ceruloplasmin*	Aceruloplasminemia	Oxidation of Fe^2+^ to Fe^3+^; trafficking and translocation of iron.	Oxidative stress; lipid peroxidation; cell death.
*Ferritin light chain* (ferritin subunit)	Neuroferritinopathy	Iron binding and storage.	Oxidative stress; lipid peroxidation; decrease of ferritin receptor level; ferritin aggregation.
*Phospholipase A2 group IV* (Ca^2+^-independent phospholipase A2β)	PLA2G6-Associated Neurodegeneration	Hydrolysis of membrane phospholipids.	Lipid peroxidation; alteration of phospholipids metabolism; impairment of membranes dynamics; accumulation of ubiquitinated proteins, α-synuclein and phosphorylated tau, mitochondrial dysfunctions.
*Fatty acid 2-hydroxylase*	Fatty Acid Hydroxylase-Associated Neurodegeneration	Generation of 2-hydroxylated phospholipids.	Demyelination and degeneration of neuronal membranes; impairments of autophagy; mitochondrial morphology alterations.
*Pantothenate kinase 2*	Pantothenate Kinase-Associated Neurodegeneration	Phosphorylation of pantothenate (Coenzyme a biosynthesis).	Oxidative stress; lipid peroxidation; energy imbalance; increased apoptosis; alteration of mitochondrial membrane potential; decreased mitochondrial respiration; cell death.
*Coenzyme A Synthase*	COASY Protein-Associated Neurodegeneration	Coenzyme a biosynthesis.	Oxidative stress; lipid dyshomeostasis; decreased mitochondrial respiration.
*C19orf12*	Mitochondrial Membrane Protein-Associated Neurodegeneration	Putative function in the response to oxidative stress at the mitochondrial level.	Mitochondrial fragmentation; reduced oxygen consumption; lipid alterations; autophagic defects; accumulation of α-synuclein and phosphorylated tau.
*WD repeat domain 45*	β-propeller protein-associated neurodegeneration	Binding with phosphatidylinositol-3-phosphate. Autophagosomes formation, expansion and closure.	Mitochondrial morphology alterations; increased apoptosis; autophagic flux dysfunctions.
*ATP13A*	Kufor–Rakeb disease	Translocation of ions and polyamines across the lysosomal membrane.	Oxidative stress; mitochondrial fragmentation; autophagic defects; accumulation of insoluble proteins and organelles.
*DCAF17*	Woodhouse–Sakati syndrome	Nucleolar protein of unknown function.	DNA methylation, alterations of cell cycle progression; increased apoptosis.

## Data Availability

Not applicable.
